# Efficacy and safety of direct oral anticoagulants versus low-molecular-weight heparin for thromboprophylaxis after cancer surgery: a systematic review and meta-analysis

**DOI:** 10.1186/s12957-024-03341-5

**Published:** 2024-02-26

**Authors:** Hong Zhou, Ting-Ting Chen, Ling-ling Ye, Jun-Jie Ma, Jin-Hua Zhang

**Affiliations:** 1 Department of Pharmacy, Fujian Maternity and Child Health Hospital College of Clinical Medicine for Obstetrics & Gynecology and Pediatrics, Fujian Medical University, Fuzhou, 350001 Fujian China; 2 School of Medicine, Huaqiao University, Quanzhou, 362021 Fujian China

**Keywords:** Cancer surgery, Direct oral anticoagulants, Low-molecular-weight heparin, Meta-analysis, Thromboprophylaxis

## Abstract

**Background:**

Direct oral anticoagulants (DOACs) used as an alternative to low-molecular-weight heparin (LMWH) for thromboprophylaxis after cancer surgery for venous thromboembolic events (VTE) remains unclear. This study aimed to investigate the efficacy and safety of DOACs versus LMWH in these patients.

**Materials and methods:**

A search of EMBASE, MEDLINE, Cochrane Central Register of Controlled Trials (CENTRAL), and Web of Science was carried out and included all randomized controlled trials (RCTs) and observational studies that directly compared DOACs with LMWH for thromboprophylaxis in patients after cancer surgery through July 25, 2023. The primary efficacy and safety outcomes were VTE, major bleeding, and clinically relevant non-major bleeding (CRNMB) within 30 days of surgery. The risk of bias was assessed using the Cochrane Risk of Bias 2 (RoB2) tool for RCTs and ROBINS-I tool for non-randomized studies. This study was registered in PROSPERO (CRD42023445386).

**Results:**

We retrieved 5149articles, selected 27 for eligibility, and included 10 studies (three RCTs and seven observational studies) encompassing 3054 patients who underwent postoperative thromboprophylaxis with DOACs (41%) or LMWH (59%). Compared to LMWH thromboprophylaxis, DOACs had a comparable risk of VTE (RR:0.69[95% CI:0.46–1.02], I^2^ = 0%), major bleeding (RR:1.55 [95% CI:0.82–2.93], I^2^ = 2%), and CRNMB (RR, 0.89 [95% CI, 0.4–1.98], I^2^ = 31%) during the 30-day postoperative period. Subgroup analysis of VTE and major bleeding suggested no differences according to study type, extended thromboprophylaxis, tumor types, or different types of DOAC.

**Conclusion:**

DOACs are potentially effective alternatives to LMWH for thromboprophylaxis in patients undergoing cancer surgery, without increasing the risk of major bleeding events.

**Supplementary Information:**

The online version contains supplementary material available at 10.1186/s12957-024-03341-5.

## Introduction

Venous thromboembolic events (VTEs), including deep vein thrombosis (DVT) and pulmonary embolism (PE), remain major causes of morbidity and mortality in patients with cancer [1]. Patients with cancer increased sevenfold risk of venous thrombosis compared with non-cancer patients (odds ratio [OR], 6.7; 95% confidence interval [CI], 5.2–8.6) [2]. Surgical trauma increases the risk of developing VTE. This increased twofold risk of VTE in patients with known cancer vs. non-cancer patients undergoing the same surgery [3]. Education for the risk assessment and prophylaxis of VTE and considering guidelines are important for making the optimal thromboprophylaxis decision [4]. Current guidelines [5–8] recommend the use of VTE prophylaxis with 7–10 days of low-molecular-weight heparin (LMWH) or unfractionated heparin in patients who underwent cancer-related surgery, and 4 weeks extended-duration LMWHs prophylaxis for abdominal-pelvic surgery because of the significantly reduced incidence of VTE without increasing bleeding complications or mortality [9, 10].

However, the use of subcutaneous low-molecular-weight heparin has some limitations such as injection site reaction, pain, bruising, and bleeding, which may impair the quality of life of patients [7]. Patients taking apixaban demonstrated good adherence, with significantly increased adherence from 3 to 25% compared with enoxaparin [11]. Currently, the use of DOACs as an effective and safe option for the treatment of cancer-associated thrombosis in selected cancer patients has been supported by the results of several high-quality randomized controlled trials (RCTs) [6, 12–16]and is strong recommended in guidelines [17–20]. However, evidence to support the use of direct oral anticoagulants as an alternative to LMWH for the prophylaxis of postoperative VTE in patients with cancer is insufficient. Recently, three randomized clinical trials showed evidence for the safety and efficacy of two direct oral anticoagulants for extended thromboprophylaxis of malignant neoplasms after surgery [5, 21, 22], and apixaban and rivaroxaban were weakly recommended as options for extended pharmacological thromboprophylaxis after cancer surgery [17].

Therefore, we present the results of a systematic review and meta-analysis of all RCTs and observational studies comparing the efficacy and safety of DOACs and LMWH for postoperative VTE prophylaxis in cancer patients undergoing surgery.

## Materials and methods

This work was reported in line with the PRISM, Supplementary file 1 (Preferred Reporting Items for Systematic Reviews and Meta-Analysis)2020 [23] and AMSTA, Supplementary file 2 (Assessing the Methodological Quality of systematic reviews) Guidelines [24]. The systematic review protocol and search strategy were registered in the PROSPERO International Prospective Register of Systematic Reviews (ID number and hyperlink: CRD42023445386).

### Search strategy

We conducted a systematic literature search using EMBASE (1947 to July 25, 2023), MEDLINE via PubMed (1946 to July 25, 2023), the Cochrane Central Register of Controlled Trials (CENTRAL, searched July 25, 2023), and Web of Science (1985 to July 25, 2023), and searched www.clinicaltrials.gov for completed and ongoing research, as well as references of narrative reviews, and included trials from all languages through July 25, 2023. The complete search strategy is available in Supplementary Table 1.

### Inclusion and exclusion criteria

We included articles that included conference abstracts if they met the following criteria: (1) randomized controlled trials (RCTs) and observational studies; (2) adult patients (18 years old or older) who underwent cancer-related surgery; (3) directly compared DOAC (dabigatran, rivaroxaban, apixaban, betrixaban, or edoxaban) to LMWH (dalteparin, enoxaparin, tinzaparin, or nadroparin) for thromboprophylaxis; and (4) reported primary efficacy or safety outcomes. The meta-analysis excluded case reports, review articles, descriptive articles, animal trials, non-cancer surgery, non-comparative observational studies, not DOACs vs. LMWH, and lacking the outcomes of interest.

### Outcome measures

The primary efficacy outcome was VTE, defined as asymptomatic or symptomatic DVT of the lower extremity with or without PE, reported within the 30-day postoperative period. The primary safety outcome was major bleeding and clinically relevant non-major bleeding (CRNMB), defined according to the International Society on Thrombosis and Haemostasis [8, 25].

### Data extraction and risk of bias assessment

Two investigators (HZ and TTC) independently selected the title and abstract, and extracted the data. Discrepancies were resolved by consensus and were reviewed by a third investigator. The quality of RCTs was identified using the Cochrane Risk of Bias 2 (RoB2) tool [26] and observational studies were assessed using the Risk of Bias in Non-randomized Studies (ROBINS-I) Tool [27] independently by two investigators (HZ and TTC). The RoB2 tool assesses five domains: adequacy of the randomization process, deviations from intended interventions, missingness of outcome data, measurement of the outcome, and selection of the reported result. The ROBINS-I tool assesses seven domains: confounding, selection of participants, classification of intervention, deviations from intended intervention, missing data, measurement of outcomes, and selection of the reported result.

### Statistical analysis

Forest plots of comparative relative risk (RR) and 95% confidence of primary efficacy and safety outcomes were calculated and pooled using the Mantel–Haenszel random effects model in Revman 5.3 software [28]. Heterogeneity across the trials was assessed using Cochran's Q test and the I^2^ statistic [29]. Subgroup analyses were performed according to study design (RCTs versus observational studies), extended thromboprophylaxis, and different tumor types. Sensitivity analyses were conducted to determine the robustness of the results by using the leave-one-out method. We did not evaluate publication bias because fewer than ten studies reported primary efficacy or safety outcomes [30].

## Results

### Study selection and characteristics

**Fig. 1:**
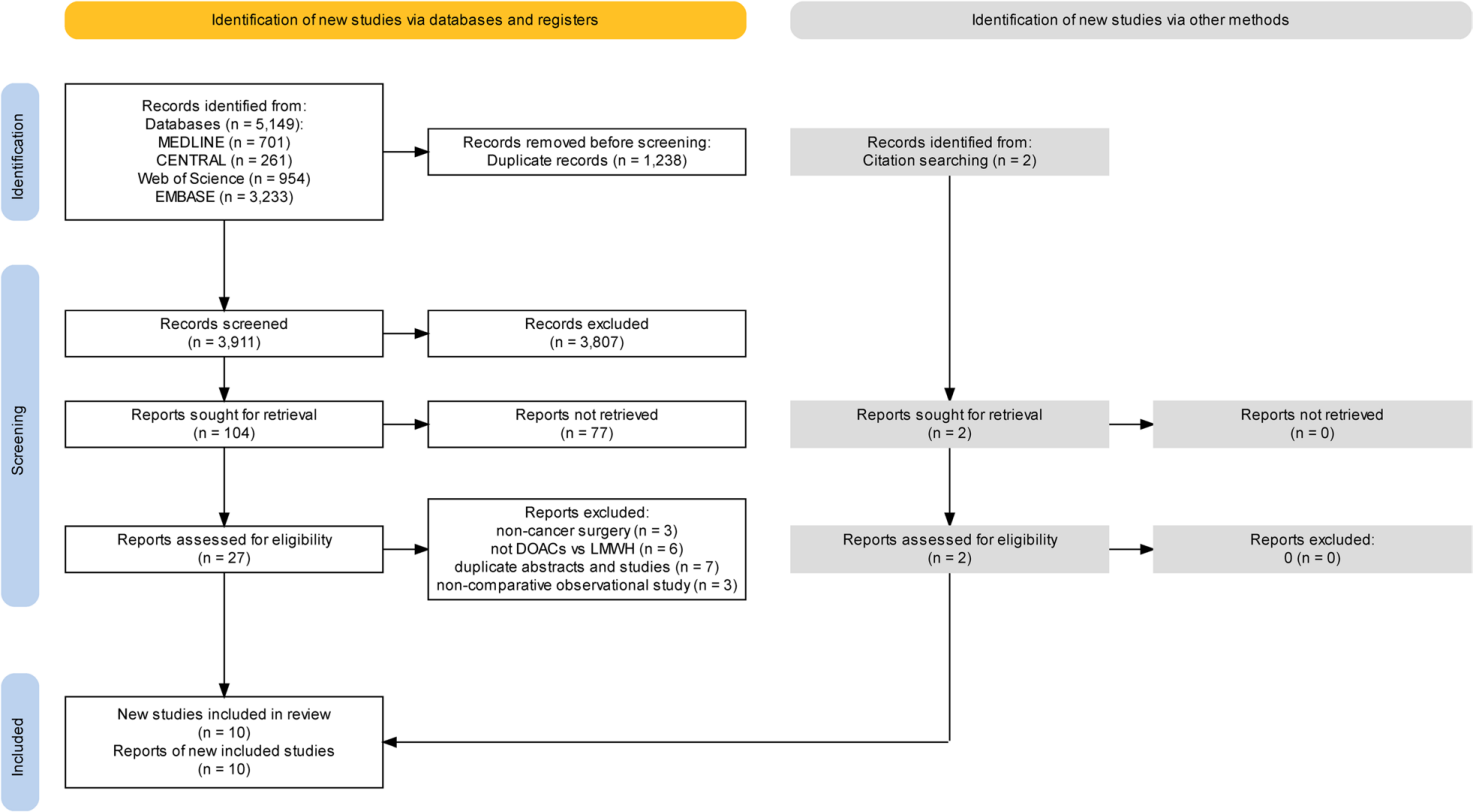
PRISMA flowchart

A total of 5149 articles were identified and screened for titles and abstracts, and 27 full-text articles were selected for eligibility. A detailed screening process is presented below in the form of a PRISMA flow diagram produced by the tool [31] (Fig. 1). A total of 10 studies (three RCTs [21, 22, 32] and 7observational studies [33–39]), encompassing 3054patients, were included in the systematic review. Of the 10 studies, eight (three RCTs [21, 22, 32] and five observational studies [34, 36–39]) were included in the pooled analysis comparing the efficacy and safety of thromboprophylaxis after cancer surgery within 30 days. The types of cancer included gynecological malignancies (*n* = 5), urological malignancies (*n* = 3), Pancreatic adenocarcinoma (*n* = 1) and lung cancer (*n* = 1). The included studies used rivaroxaban (*n* = 4), apixaban (*n* = 4), and dabigatran (*n* = 1); one observational study used these three drugs. The characteristics of the included studies are summarized in Table 1.

**Table 1:** Summary of studies from systematic review of DOAC vs. LMWH for thromboprophylaxis after cancer-related surgery

Study	Publication Type	Design	Median age(years)^a^	Male^a^%	Malignancy Type	Study Size^a^	Intervention		Outcomes		
							DOAC	LMWH	Endpoint (time)	DOAC	LMWH
Guntupalli 2020 [21]	Full-Text	RCT	58/58.5	0	Gynecologic malignancy	204/196	Apixaban	Enoxaparin	VTE (90 days) MB(30 days) CRNMB(30 days) Readmission (30 days)	1%(2/204) 0.5%(1/204) 5.9%(12/204) 2.9%(6/204)	1.5%(3/196) 0.5%(1/196) 9.7%(19/196) 2.6%(5/196)
Oliveira 2022 [22]	Full-Text	RCT	54/56	0	Gynecologic malignancy	114/114	Rivaroxaban	Enoxaparin	VTE(30 days) MB(30 days) CRNMB(30 days)	3.5%(4/114) 0%(0/114) 0%(0/114)	4.4%(5/114) 0%(0/114) 2.6%(3/114)
Zhao 2023 [32]	Full-Text	RCT	61.2/61.7	53.5/47.3^b^	Lung cancer	200/203	Rivaroxaban	nadroparin	VTE(30 days) MB(30 days) CRNMB(30 days)	12.5%(25/200) 9.7%(19/196) 2.5%(5/196)	17.7%(36/203) 6.5%(13/201) 0.5%(1/201)
Nagy 2018 [33]	Abstract	Observational study	NR	0	Gynecologic malignancy	147/451	Rivaroxaban	LMWH	VTE (90 days) bleeding complications(NR)	0.7%(1/147) 2%( 3/147)	2.4%(11/451) 0.7%( 3/451)
Spénard 2023 [37]	Full-Text	Observational study	60/63	0	Gynecologic malignancy	112/144	Apixaban	Enoxaparin	VTE(30 days) MB(30 days) Readmission (30 days)	3%(3/112) 0%(0/112) 6%(7/112)	4%(6/144) 0%(0/144) 5%(7/144)
Swaroop 2021 [38]	Abstract	Observational study	NR	0	Gynecologic malignancy	82/233	Rivaroxaban	LMWH	VTE (30 days) VTE (90 days) MB (30 days)	1.2%(1/82) 2.4%(2/82) 3.7%(3/82)	1.7%(4/233) 4.3%(10/233) 0.4%(1/233)
Rashid 2018 [35]	Full-Text	Observational study	NR	59	Pancreatic adenocarcinoma	87/12	dabigatran	Enoxaparin	MB (90 days) Minor bleeding(90 days)	4.5%(4/87) 2.3%(2/87)	0%(0/12) 0%(0/12)
Rich 2023 [36]	Full-Text	Observational study	69/69	82/76	Bladder cancer	124/250	Apixaban	Enoxaparin	VTE (30 days) MB (30 days) Readmission (30 days) Mortality (30 days)	1.6%(2/124) 0.8%(1/124) 20%(25/124) 0.8%(1/124)	3.2%(8/250) 0.4%(1/250) 19%(48/250) 1.6%(4/250)
Westerman 2022 [39]	Full-Text	Observational study	66/66	84/79	Urological malignancy	154/161	Apixaban	Enoxaparin	VTE(30 days) MB(30 days) CRNMB(30 days)	0%(0/154) 0%(0/154) 3.9%(6/154)	1.7%(3/161) 1.2%(2/161) 4.3%(7/161)
Ortiz 2021 [34]	Full-Text	Observational study	65/68	69/70	Bladder cancer	29/37	Rivaroxaban (83%), Apixaban(14%) and Dabigatran(3%)	Enoxaparin	VTE(30 days) VTE(90 days) CRNMB(30 days) Readmission (30 days)	0%(0/29) 3.4%(1/29) 3.4%(1/29)	0%(0/37) 8.1%(3/37) 0%(0/37)

*RCT* randomized controlled trial, *NR* not reported, *VTE* venous thromboembolism, *MB* major bleeding, *CRNMB* clinically relevant non-major bleeding

^a^Presented as DOAC/LMWH groups

^b^Median

### Risk of bias

**Fig. 2:**
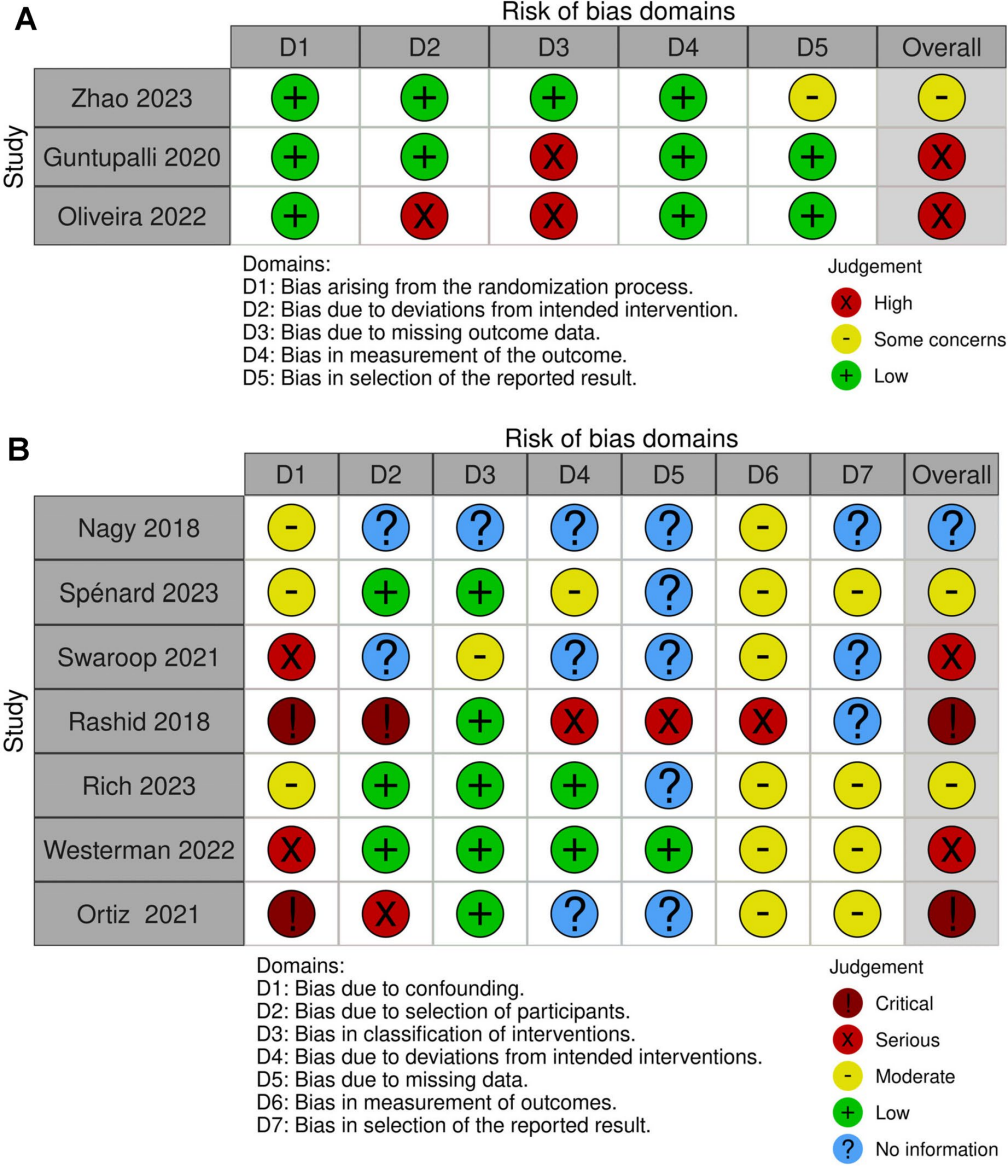
Risk of bias assessment. **A** Risk of bias for randomized controlled trials, **B** Risk of bias for observational studies

Two of the three randomized controlled trials were adjudicated to a low risk of bias [21, 32] and one had some concern because the trial was stopped due to a lower-than-expected event rate [22] (Fig. 2A). All seven observational studies had at least a moderate risk of bias due to confounding of the effect of intervention in this study, such as the type and duration of surgery, age and the presence of other VTE risks. 3 observational studies [33, 36, 37] were adjudicated to a moderate risk of confounding bias by using a multivariate logistic regression analysis method that controlled for the confounding domain (Fig. 2B). Most observational studies had a bias of missing data [33, 34, 36–39]; however, one study that used a modified intention-to-treat analysis for compliance was adjudicated to a low risk of bias due to missing data [39] (Fig. 2B).Risk-of-bias plots were created by the tool [40].

### Efficacy and safety outcomes

#### Primary efficacy outcome

**Fig. 3:**
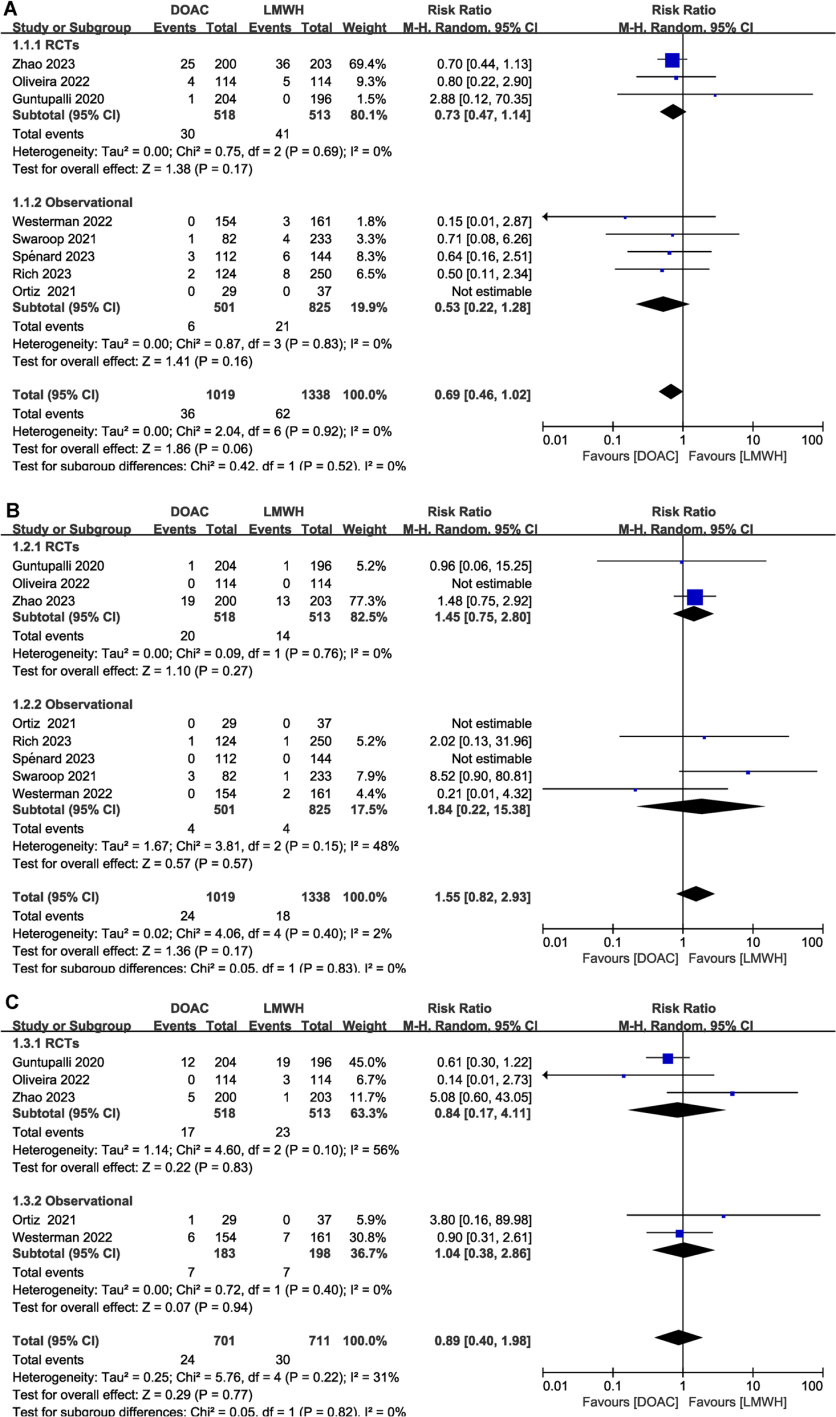
Forest plots of relative risks (RRs) for pooled outcome by 30 days postoperative comparisons between DOAC and LMWH, stratified by study design. **A** VTE, **B** Major bleeding, **C** Clinically relevant non-major bleeding (CRNMB)

Of the 10 studies, 30-day clinical VTE was assessed in 8 studies (3 RCTs [21, 22, 32] and 5 observational studies [34, 36–39]). We pooled the outcome by 30 days postoperative comparisons between DOAC and LMWH. During the 30-day postoperative period, DOACs (36/1019) had a comparable incidence of VTE when compared to LMWH (62/1338) (3.5% vs. 4.6%, RR:0.69[95% CI:0.46–1.02], *P* value for Cochran Q = 0.92, I^2^ = 0%; Fig. 3A). Meanwhile, we also pooled the data from 4 studies (1 RCT [20] and 3 observational studies [32, 33, 37]) and showed no significant difference between both groups for postoperative VTE within 90 days (Supplementary Fig. 1A).

#### Primary safety outcome

30-day major bleeding was reported in 8 studies (3 RCTs [21, 22, 32] and 5 observational studies [34, 36–39]). There was no statistically significant difference in the incidence of major bleeding with DOAC (24/1019) compared with LMWH (18/1338) (2.4% vs. 1.3%, RR: 1.55 [95% CI: 0.82–2.93], Cochran Q = 0.4, I^2^ = 2%; Fig. 3B). The result of 90-day major bleeding pooled data from 3 studies (1 RCT [20] and 2 observational studies [34, 37]) were consistent with those above (Supplementary Fig. 1B). CRNMB was reported in 5 studies (3 RCTs [21, 22, 32] and 2 observational studies [34, 39]). DOACs had a comparable risk of CRNMB when compared to LMWH (3.4% vs. 4.2%, RR, 0.89 [95% CI, 0.4–1.98], *P* value for Cochran Q = 0.22, I^2^ = 31%; Fig. 3C).

#### Subgroup analysis

We conducted a subgroup analysis of 30-day VTE and major bleeding according to the study type (RCTs versus observational studies), duration of thromboprophylaxis (extended versus non-extended), tumor type (gynecologic malignancy, urological malignancy, and lung cancer), and different types of DOAC. The results showed no significant differences and were summarized in Supplementary Fig. 2A–4B. In addition, sensitivity analyses were conducted to show no significant influence on the results of sequential removal of each study (Supplementary Table 2).

## Discussion

To the best of our knowledge, this is the first systematic review and meta-analysis to directly compare the effectiveness and safety of DOACs and LMWH for thromboprophylaxis in postoperative patients by combining RCTs and observational studies. In this systematic review and meta-analysis, DOACs and LMWH for thromboprophylaxis seemed to have similar efficacy and safety profiles in terms of subsequent venous thromboembolism and clinically relevant bleeding complications. The results did not seem to differ between RCTs and observational studies, extended thromboprophylaxis, or tumor type.

The degree of VTE risk in patients undergoing cancer surgery varies according to the type and duration of surgery, immobilization status of the patient and the presence of other VTE risks in the cancer surgery patient [4]. In our meta-analysis, there was a low 30-day postoperative rate of VTE between the DOAC and LMWH groups (3.5% vs. 4.6%), which is similar to that previously reported in other cancer-related surgery trials [21, 22]. One RCT of lung cancer with non-extended prophylaxis included in the studies showed a higher incidence of VTE and MB than other studies of abdominopelvic cancer with extended prophylaxis. However, subgroup analysis suggested no difference according to the duration of thromboprophylaxis (extended or non-extended) and tumor type (gynecologic malignancy, urological malignancy, and lung cancer). Lung cancer is associated with a higher risk of VTE than other malignant solid organ tumors [41, 42]. Extended thromboprophylaxis reduced the tenfold risk of pulmonary embolism in patients who underwent resection of primary lung cancer and was independently associated with a reduction in postoperative PE [43]. Therefore, DOACs might be an efficacious alternative to LMWH for extended thromboprophylaxis to reduce risk of VTE in patients undergoing lung cancer resection surgery, and further studies are warranted.

Regarding the safety of major bleeding and CRNMB, no statistical significance was found in our meta-analysis. Previous studies have shown that patients with gastrointestinal cancer have a high rate of major hemorrhage [44, 45]. However, updated meta-analyses of randomized trials found that major bleeding occurred more frequently with DOACs, but there was no difference in the risk of overall major bleeding between DOACs and LMWH for cancer-related venous thromboembolism [46–48].

The results of the present study should be interpreted with caution because of the following limitations. First, the number of three RCTs in the meta-analysis was small, and seven of the ten included studies were observational studies, which may have introduced bias. However, the subgroup analysis suggested no differences between RCTs and observational studies, and the outcomes of observational studies were consistent with those of RCTs. Second, the types of medications used were mainly apixaban and rivaroxaban. Future studies are encouraged to investigate other DOACs used in VTE prophylaxis in cancer-related surgery. Therefore, we used a subgroup analysis to reduce the impact of these potential limitations. Thirdly, although the long-term effect of DOAC versus LMWH in postoperative thromboprophylaxis is consistent with the 30-day effect, there are few included studies and more RCTs of long-term effects are needed. Lastly, the tumor type was mainly gynecologic malignancy and urological malignancy, and additional evidence is expected for gastrointestinal malignancies and other malignant tumors.

## Conclusion

DOACs are equivalent to LMWH in preventing postoperative VTE as thromboprophylaxis after cancer-related surgery. These findings suggest that oral DOACs (apixaban and rivaroxaban) are potentially effective and safe alternatives to subcutaneous LMWH for thromboprophylaxis in patients undergoing cancer surgery. Further studies are needed for thromboprophylaxis in patients with gastrointestinal malignancies and other tumors undergoing surgery.

### Supplementary Information

**Supplementary Material 1.**

**Supplementary Material 2.**

**Supplementary Material 3.**

## Data Availability

This is a meta-analysis article, and data availability is not applicable. Please contact the corresponding author if data are needed.

## References

[1] Kekre N, Connors JM (2019). Venous thromboembolism incidence in hematologic malignancies. Blood Rev.

[2] Blom JW, Doggen CJM, Osanto S, Rosendaal FR (2005). Malignancies, prothrombotic mutations, and the risk of venous thrombosis. JAMA.

[3] Behranwala KA, Williamson RCN (2009). Cancer-associated venous thrombosis in the surgical setting. Ann Surg.

[4] Kiracı ZK, Yalçın N, Cennet Ö, Demirkan K, Yorgancı K (2023). Education and clinical pharmacist-led management strategies for the risk and prophylaxis of venous thromboembolism in general surgery. Thromb J.

[5] Becattini C, Pace U, Pirozzi F (2022). Rivaroxaban vs placebo for extended antithrombotic prophylaxis after laparoscopic surgery for colorectal cancer. Blood.

[6] Young AM, Marshall A, Thirlwall J (2018). Comparison of an Oral Factor Xa Inhibitor With Low Molecular Weight Heparin in Patients With Cancer With Venous Thromboembolism: Results of a Randomized Trial (SELECT-D). J Clin Oncol.

[7] Khorana AA, McCrae KR, Milentijevic D (2017). Current practice patterns and patient persistence with anticoagulant treatments for cancer-associated thrombosis. Res Pract Thromb Haemost.

[8] Schulman S, Kearon C (2005). Definition of major bleeding in clinical investigations of antihemostatic medicinal products in non-surgical patients. J Thromb Haemost.

[9] Felder S, Rasmussen MS, King R (2019). Prolonged thromboprophylaxis with low molecular weight heparin for abdominal or pelvic surgery. Cochrane Database Syst Rev.

[10] Knoll W, Fergusson N, Ivankovic V (2021). Extended thromboprophylaxis following major abdominal/pelvic cancer-related surgery: a systematic review and meta-analysis of the literature. Thromb Res.

[11] Ross ME, Glickman A, Brennecke A, Tayebnejad A, Guntupalli SR (2020). Adherence to postoperative thromboprophylactic medication among gynecologic oncology patients: a subanalysis. Gynecol Oncol.

[12] Schrag D, Uno H, Rosovsky R (2023). Direct oral anticoagulants vs low-molecular-weight heparin and recurrent VTE in patients with cancer: a randomized clinical trial. JAMA.

[13] Planquette B, Bertoletti L, Charles-Nelson A (2022). Rivaroxaban vs dalteparin in cancer-associated thromboembolism: a randomized trial. Chest.

[14] McBane RD, Wysokinski WE, Le-Rademacher JG (2020). Apixaban and dalteparin in active malignancy-associated venous thromboembolism: the ADAM VTE trial. J Thromb Haemost.

[15] Agnelli G, Becattini C, Meyer G (2020). Apixaban for the treatment of venous thromboembolism associated with cancer. N Engl J Med.

[16] Raskob GE, van Es N, Verhamme P (2018). Edoxaban for the treatment of cancer-associated venous thromboembolism. N Engl J Med.

[17] Key NS, Khorana AA, Kuderer NM (2023). Venous thromboembolism prophylaxis and treatment in patients with cancer: ASCO guideline update. J Clin Oncol.

[18] Falanga A, Ay C, Di Nisio M (2023). Venous thromboembolism in cancer patients: ESMO Clinical Practice Guideline. Ann Oncol.

[19] Farge D, Frere C, Connors JM (2022). 2022 international clinical practice guidelines for the treatment and prophylaxis of venous thromboembolism in patients with cancer, including patients with COVID-19. Lancet Oncol.

[20] Lyman GH, Carrier M, Ay C (2021). American Society of Hematology 2021 guidelines for management of venous thromboembolism: prevention and treatment in patients with cancer. Blood Adv.

[21] Guntupalli SR, Brennecke A, Behbakht K (2020). Safety and efficacy of apixaban vs enoxaparin for preventing postoperative venous thromboembolism in women undergoing surgery for gynecologic malignant neoplasm: a randomized clinical trial. JAMA Netw Open.

[22] Longo de Oliveira ALM, de Oliveira Pereira RF, Agati LB (2022). Rivaroxaban Versus Enoxaparin for Thromboprophylaxis After major Gynecological Cancer Surgery: The VALERIA Trial: Venous thromboembolism prophylAxis after gynecoLogical pElvic cancer surgery with RIvaroxaban versus enoxAparin (VALERIA trial). Clin Appl Thromb Hemost..

[23] Page MJ, McKenzie JE, Bossuyt PM (2021). The PRISMA 2020 statement: an updated guideline for reporting systematic reviews. Int J Surg (London, England).

[24] Shea BJ, Reeves BC, Wells G (2017). AMSTAR 2: a critical appraisal tool for systematic reviews that include randomised or non-randomised studies of healthcare interventions, or both. BMJ (Clinical research ed).

[25] Kaatz S, Ahmad D, Spyropoulos AC, Schulman S (2015). Definition of clinically relevant non-major bleeding in studies of anticoagulants in atrial fibrillation and venous thromboembolic disease in non-surgical patients: communication from the SSC of the ISTH. J Thromb Haemost.

[26] Sterne JAC, Savović J, Page MJ (2019). RoB 2: a revised tool for assessing risk of bias in randomised trials. BMJ (Clinical Research ed).

[27] Sterne JA, Hernán MA, Reeves BC (2016). ROBINS-I: a tool for assessing risk of bias in non-randomised studies of interventions. BMJ (Clinical research ed).

[28] DerSimonian R, Laird N (1986). Meta-analysis in clinical trials. Control Clin Trials.

[29] Higgins JPT, Thompson SG, Deeks JJ, Altman DG (2003). Measuring inconsistency in meta-analyses. BMJ (Clinical research ed).

[30] Egger M, Davey Smith G, Schneider M, Minder C (1997). Bias in meta-analysis detected by a simple, graphical test. BMJ (Clinical research ed).

[31] Haddaway NR, Page MJ, Pritchard CC, McGuinness LA (2022). PRISMA2020: an R package and Shiny app for producing PRISMA 2020-compliant flow diagrams, with interactivity for optimised digital transparency and Open Synthesis. Campbell Syst Rev.

[32] Zhao M, Bao Y, Jiang C (2023). Rivaroxaban versus nadroparin for thromboprophylaxis following thoracic surgery for lung cancer: a randomized, noninferiority trial. Am J Hematol..

[33] Nagy A, Tegge AN, Borden LE, Osborne JL, Valea FA, Iglesias DA (2018). A retrospective comparison of oral rivaroxaban versus subcutaneous low-molecular-weight heparin for postoperative thromboprophylaxis in women with a gynecologic malignancy. Gynecol Oncol.

[34] Ortiz RM, Golijanin B, O'Rourke TK (2021). Direct oral anticoagulants for venous thromboembolism prophylaxis following robot-assisted radical cystectomy: a retrospective feasibility study at a single academic medical center. Urology.

[35] Rashid MF, Jackson TL, Morgan JA (2019). Dabigatran (Pradaxa) is safe for extended venous thromboembolism prophylaxis after surgery for pancreatic cancer. J Gastrointest Surg.

[36] Rich JM, Elkun Y, Geduldig J (2023). Outcomes from a prospectively implemented protocol using apixaban after robotic radical cystectomy. BJU Int..

[37] Spénard E, Geerts W, Lin Y (2023). Apixaban for extended postoperative thromboprophylaxis in gynecologic oncology patients undergoing laparotomy. Gynecol Oncol.

[38] Swaroop M, Borden L, Locklear T (2021). Postoperative thromboprophylaxis with oral rivaroxaban versus subcutaneous low-molecular-weight heparin: a retrospective comparison in women with a gynecologic malignancy. Gynecol Oncol.

[39] Westerman ME, Bree KK, Msaouel P (2022). Apixaban vs enoxaparin for post-surgical extended-duration venous thromboembolic event prophylaxis: a prospective quality improvement study. J Urol.

[40] McGuinness LA, Higgins JPT (2021). Risk-of-bias VISualization (robvis): An R package and Shiny web app for visualizing risk-of-bias assessments. Res Synth Methods..

[41] Merkow RP, Bilimoria KY, McCarter MD (2011). Post-discharge venous thromboembolism after cancer surgery: extending the case for extended prophylaxis. Ann Surg.

[42] Trinh VQ, Karakiewicz PI, Sammon J (2014). Venous thromboembolism after major cancer surgery: temporal trends and patterns of care. JAMA Surg.

[43] Kho J, Mitchell J, Curry N, Di Chiara F, Stavroulias D, Belcher E (2022). Should all patients receive extended thromboprophylaxis after resection of primary lung cancer?. J Thorac Cardiovasc Surg..

[44] Li A, Garcia DA, Lyman GH, Carrier M (2019). Direct oral anticoagulant (DOAC) versus low-molecular-weight heparin (LMWH) for treatment of cancer associated thrombosis (CAT): a systematic review and meta-analysis. Thromb Res.

[45] Seo S, Ryu MH, Kang YK (2016). Oral rivaroxaban versus subcutaneous low molecular weight heparin treatment for venous thromboembolism in patients with upper gastrointestinal, hepatobiliary and pancreatic cancer. Ann Oncol..

[46] Haykal T, Zayed Y, Deliwala S (2020). Direct oral anticoagulant versus low-molecular-weight heparin for treatment of venous thromboembolism in cancer patients: an updated meta-analysis of randomized controlled trials. Thromb Res.

[47] Giustozzi M, Agnelli G, Del Toro-Cervera J (2020). Direct oral anticoagulants for the treatment of acute venous thromboembolism associated with cancer: a systematic review and meta-analysis. Thromb Haemost.

[48] Elbadawi A, Shnoda M, Mahmoud K, Elgendy IY (2021). Efficacy and safety of direct oral anticoagulants vs. low molecular weight heparin for cancer-related venous thromboembolism: a meta-analysis of randomized trials. Eur Heart J Cardiovasc Pharmacother.

