# Supplementary material for: Efficacy and safety of direct oral anticoagulants versus low-molecular-weight heparin for thromboprophylaxis after cancer surgery: a systematic review and meta-analysis
Source: World J Surg Oncol. 2024 Feb 26;22:69. doi: 10.1186/s12957-024-03341-5 (PMC10895850; doi:10.1186/s12957-024-03341-5)
Supplement: Supplementary file 3 — Supplementary Material 3. [file 12957_2024_3341_MOESM3_ESM.docx]

Supplementary Material

**Supplemental Table 1**. Electronic search strategies (last done on 25/7/23)

EMBASE (via Elsevier):

- 'neoplasm'/exp OR 'carcinoma'/exp OR 'cancer'/exp OR 'malignan*' OR 'neoplasm*' OR 'cancer*' OR 'carcinoma*' OR 'tumour*' OR 'tumor*'
- 'apixaban*':ti,ab,kw OR 'betrixaban*':ti,ab,kw OR 'edoxaban*':ti,ab,kw OR 'rivaroxaban*':ti,ab,kw OR 'dabigatran*':ti,ab,kw OR 'ximelagatran*':ti,ab,kw OR 'factor xa inhibitor*':ti,ab,kw OR 'non-vitamin k antagonist oral anticoagulant*':ti,ab,kw OR 'non-vitamin k antagonist*':ti,ab,kw OR 'direct oral anticoagulant*':ti,ab,kw OR doacs:ti,ab,kw OR doac:ti,ab,kw OR 'novel oral anticoagulant*':ti,ab,kw OR 'apixaban'/exp OR 'dabigatran'/exp OR 'edoxaban'/exp OR 'rivaroxaban'/exp OR 'betrixaban'/exp OR 'ximelagatran'/exp
- 'enoxaparin*':ti,ab,kw OR 'dalteparin*':ti,ab,kw OR 'tinzaparin*':ti,ab,kw OR 'nadroparin*':ti,ab,kw OR 'LMWH*':ti,ab,kw OR 'low molecular weight heparin':ti,ab,kw OR 'low molecular weight heparin'/exp OR 'dalteparin'/exp OR 'enoxaparin'/exp OR 'nadroparin'/exp OR 'tinzaparin'/exp
- #1 AND #2 AND #3

MEDLINE (via PubMed):

- ((((malignan*[tiab] OR neoplasm*[tiab] OR cancer*[tiab] OR carcinoma*[tiab] OR tumour*[tiab] OR tumor*[tiab]) OR (Neoplasms[MeSH Terms])) OR (cancer[MeSH Terms])) OR (Carcinoma[MeSH Terms])) OR (Adenocarcinoma[MeSH Terms])
- ((((Factor Xa Inhibitors[mesh]) OR (dabigatran[mesh])) OR (rivaroxaban[mesh])) OR (apixaban*[tiab] OR betrixaban*[tiab] OR edoxaban*[tiab] OR rivaroxaban*[tiab] OR dabigatran*[tiab] OR ximelagatran*[tiab])) OR (• factor Xa inhibitor*[tiab] OR non-vitamin K antagonist oral anticoagulant*[tiab] OR non-vitamin K antagonist*[tiab] OR direct oral anticoagulant*[tiab] OR DOACs[tiab] OR DOAC[tiab] OR novel oral anticoagulant*[tiab] OR new oral anticoagulant*[tiab])
- (Heparin, Low-Molecular-Weight[mesh]) OR (Enoxaparin*[tiab] OR dalteparin*[tiab] OR tinzaparin*[tiab] OR nadroparin*[tiab] OR LMWH*[tiab] OR Low molecular weight heparin[tiab])
- #1 AND #2 AND #3

CENTRAL (via Cochrane):

- MeSH descriptor: [Neoplasms] explode all trees
- MeSH descriptor: [Carcinoma] explode all trees
- 'malignan*' OR 'neoplasm*' OR 'cancer*' OR 'carcinoma*' OR 'tumour*' OR 'tumor*'
- MeSH descriptor: [Factor Xa Inhibitors] explode all trees
- MeSH descriptor: [Dabigatran] explode all trees
- MeSH descriptor: [Rivaroxaban] explode all trees
- 'apixaban*' OR 'betrixaban*' OR 'edoxaban*' OR 'rivaroxaban*' OR 'dabigatran*' OR 'ximelagatran*'
- 'factor Xa inhibitor*' or 'non-vitamin K antagonist oral anticoagulant*' or 'non-vitamin K antagonist*' or 'direct oral anticoagulant*' or DOACs or DOAC or 'novel oral anticoagulant*' or 'new oral anticoagulant*'
- MeSH descriptor: [Heparin, Low-Molecular-Weight] explode all trees
- 'enoxaparin*' OR 'dalteparin*' OR 'tinzaparin*' OR 'nadroparin*' OR 'LMWH*' OR 'low molecular weight heparin'
- (#1 OR #2 OR #3) AND (#4 OR #5 OR #6 OR #7 OR #8) AND (#9 OR #10)

Web of science

- 'neoplasm' OR 'carcinoma' OR 'cancer' OR 'malignan*' OR 'neoplasm*' OR 'cancer*' OR 'carcinoma*' OR 'tumour*' OR 'tumor*'
- 'apixaban*' OR 'betrixaban*' OR 'edoxaban*' OR 'rivaroxaban*' OR 'dabigatran*' OR 'ximelagatran*' OR 'factor xa inhibitor*' OR 'non-vitamin k antagonist oral anticoagulant*' OR 'non-vitamin k antagonist*' OR 'direct oral anticoagulant*' OR doacs OR doac OR 'novel oral anticoagulant*' OR 'apixaban' OR 'dabigatran' OR 'edoxaban' OR 'rivaroxaban' OR 'betrixaban' OR 'ximelagatran'
- 'enoxaparin*' OR 'dalteparin*' OR 'tinzaparin*' OR 'nadroparin*' OR 'LMWH*' OR 'low molecular weight heparin' OR 'low molecular weight heparin' OR 'dalteparin' OR 'enoxaparin' OR 'nadroparin' OR 'tinzaparin'
- #1 AND #2 AND #3

**Supplementary Table 2.** Sensitivity analysis with the leave‐one‐out method for the primary efficacy outcome

| Study Omitted | RR (95% CI) |
| --- | --- |
| Guntupalli 2020 | 0.55(0.28-1.10) |
| Oliveira 2022 | 0.55 (0.26-1.19) |
| Nagy 2018 | 0.67 (0.33-1.34) |
| Spénard 2023 | 0.60 (0.28-1.27) |
| Swaroop 2021 | 0.60 (0.30-1.20) |
| Rich 2023 | 0.63 (0.31-1.32) |
| Westerman 2022 | 0.65 (0.33-1.29) |
| Ortiz 2021 | 0.61 (0.31-1.1.8) |

*RR, risk ratio; CI, confidence interval.*

A


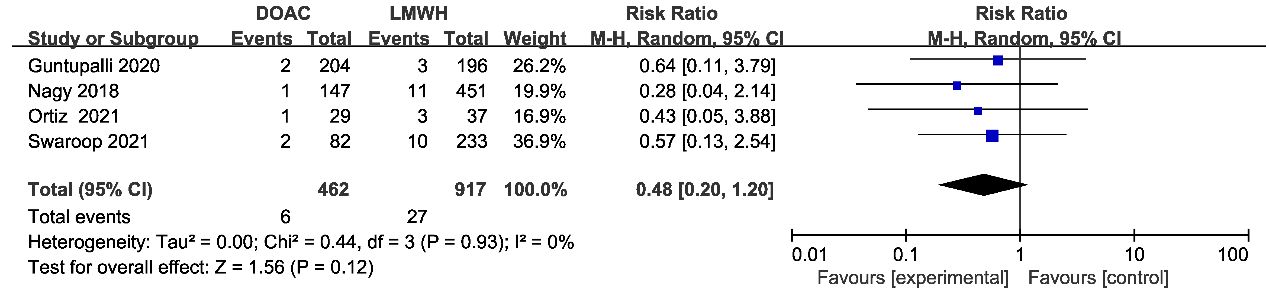


B


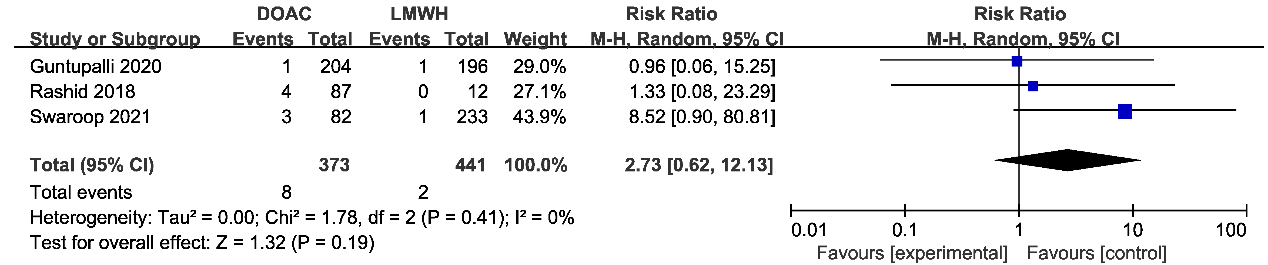


**Supplementary Figure 1.** Forest plots of relative risks (RRs) for pooled outcome by 90 days postoperative comparisons between DOAC and LMWH, stratified by study design. (A) VTE, (B) major bleeding.

A


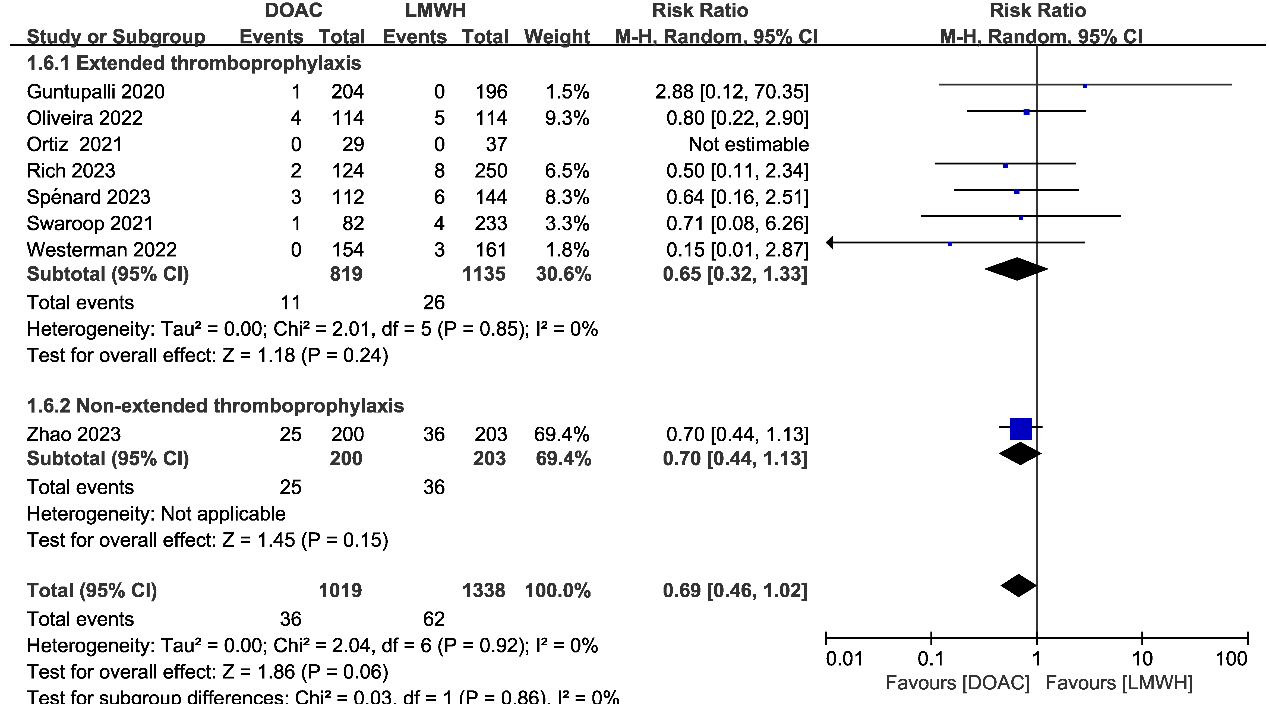


B


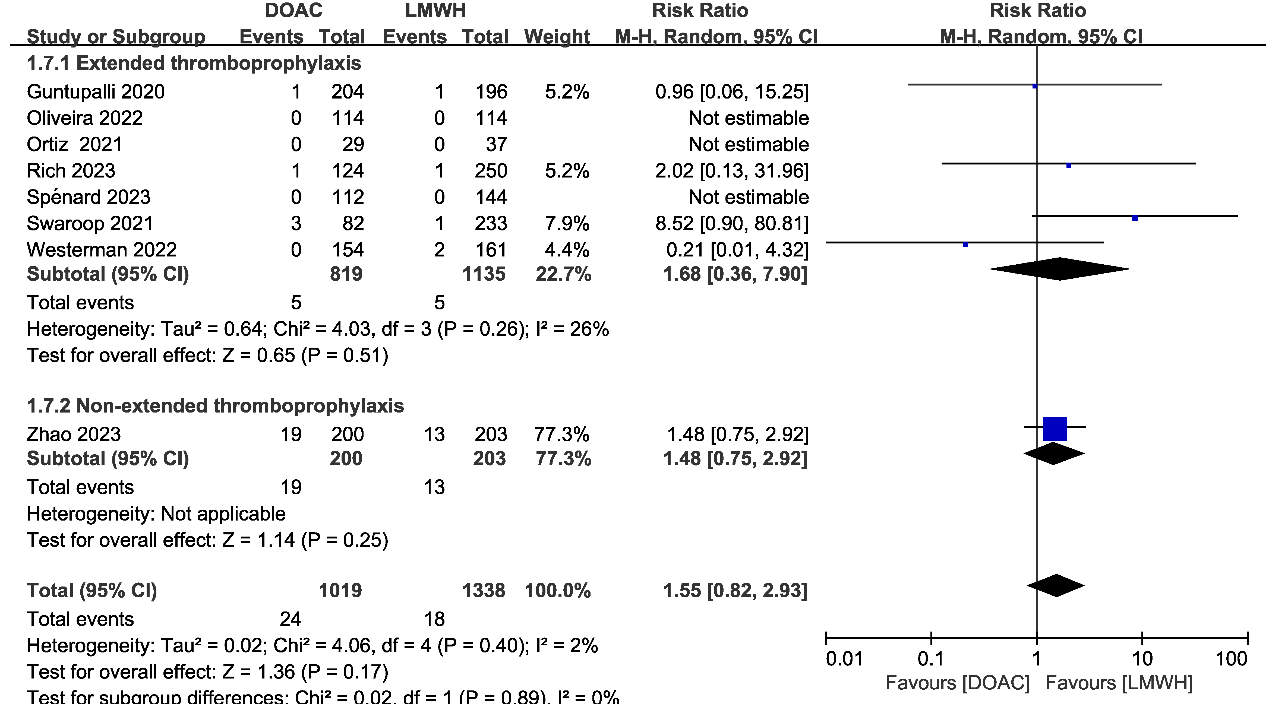


**Supplementary Figure 2.** Subgroup analyses for 30-day VTE (A) and major bleeding (B) postoperative comparisons between DOAC and LMWH for extended vs. non-extended thromboprophylaxis

A


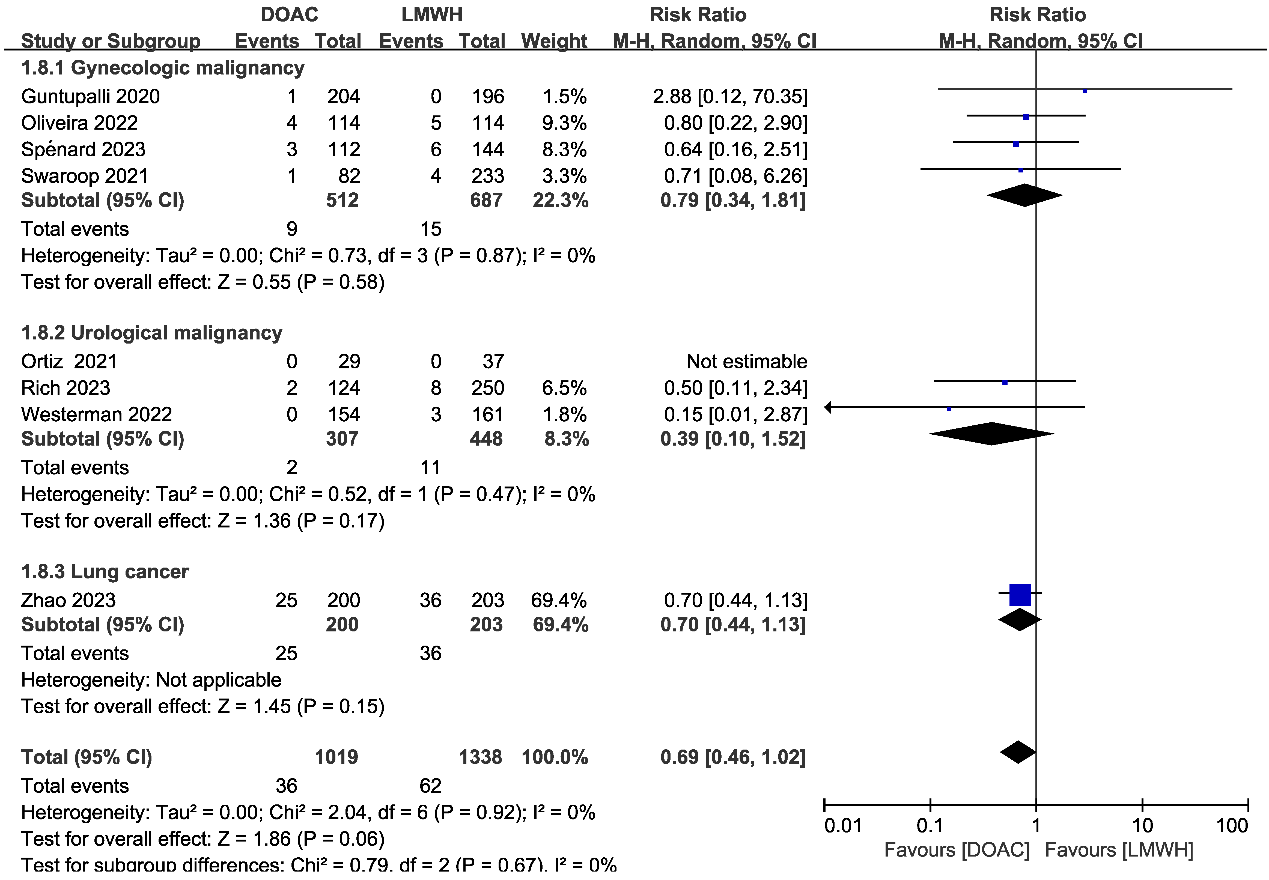


B


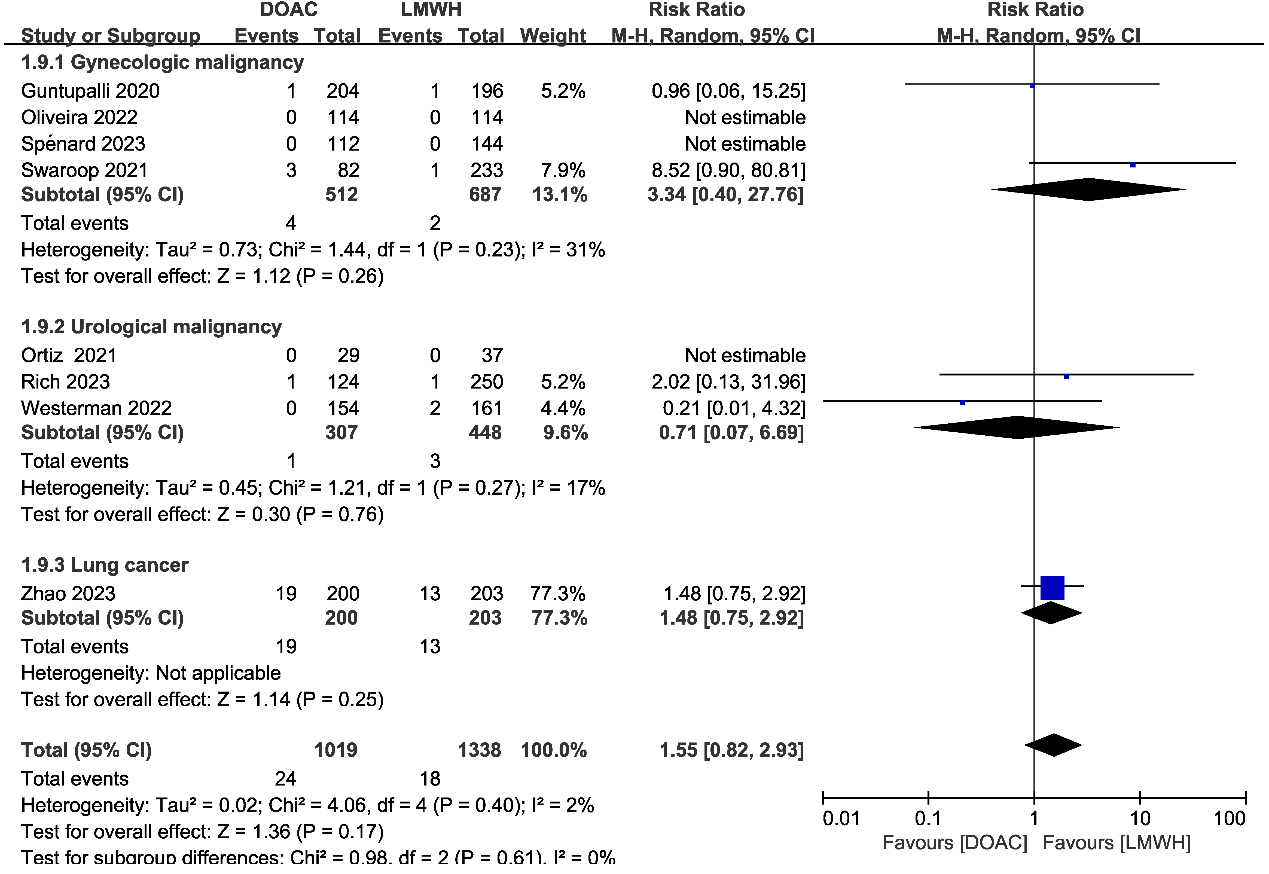


**Supplementary Figure 3.** Subgroup analyses for 30-day VTE (A) and major bleeding (B) postoperative comparisons between DOAC and LMWH for different tumor type.

A


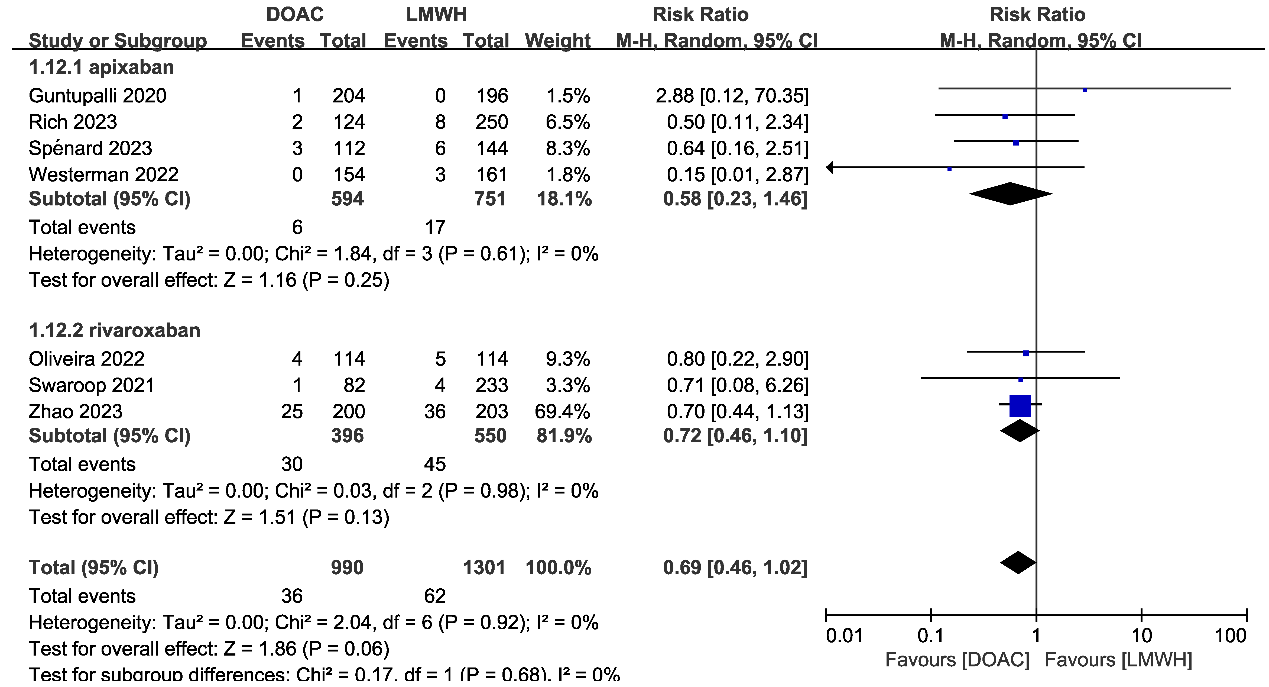


B


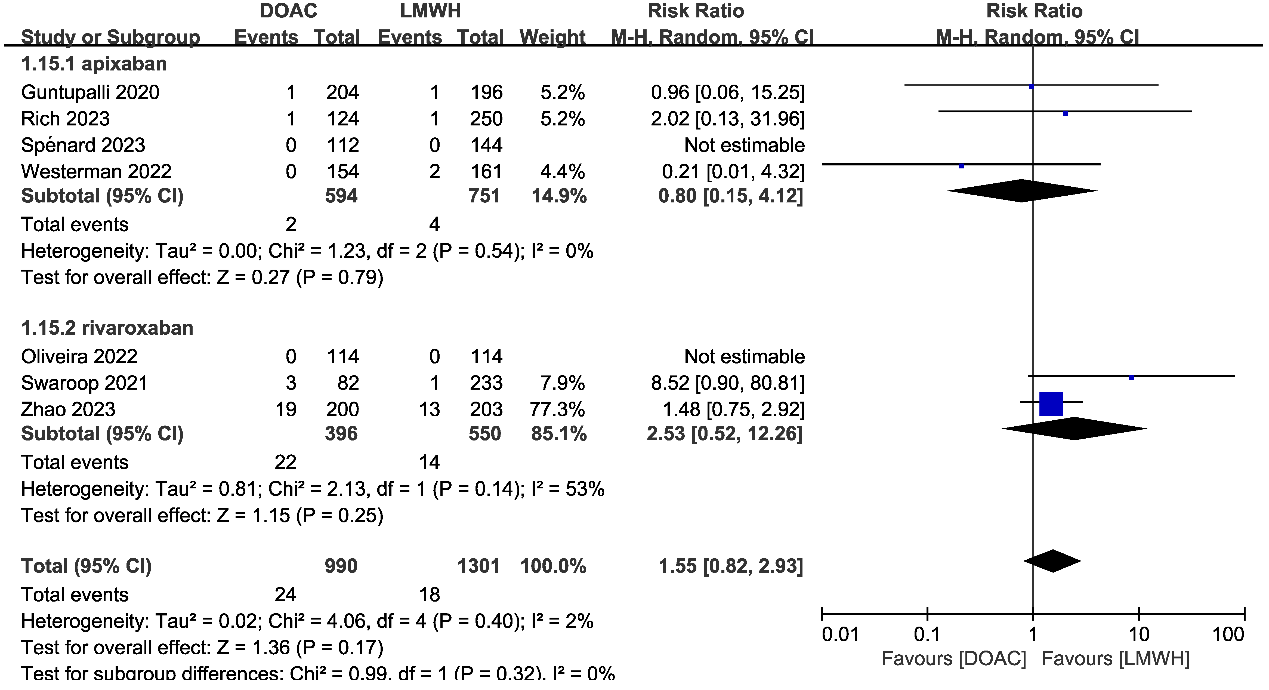


**Supplementary Figure 4.** Subgroup analyses for 30-day VTE (A) and major bleeding (B) postoperative comparisons between DOAC and LMWH for different types of DOAC.
